# The latest progress on miR‐374 and its functional implications in physiological and pathological processes

**DOI:** 10.1111/jcmm.14219

**Published:** 2019-02-17

**Authors:** Hongjun Bian, Yi Zhou, Dawei Zhou, Yongsheng Zhang, Deya Shang, Jianni Qi

**Affiliations:** ^1^ Shandong Provincial Hospital Affiliated to Shandong University Jinan Shandong China

**Keywords:** calcium handle, epilepsy, miR‐374, ncRNA, tumorigenesis

## Abstract

Non‐coding RNAs (ncRNAs) have been emerging players in cell development, differentiation, proliferation and apoptosis. Based on their differences in length and structure, they are subdivided into several categories including long non‐coding RNAs (lncRNAs >200nt), stable non‐coding RNAs (60‐300nt), microRNAs (miRs or miRNAs, 18‐24nt), circular RNAs, piwi‐interacting RNAs (26‐31nt) and small interfering RNAs (about 21nt). Therein, miRNAs not only directly regulate gene expression through pairing of nucleotide bases between the miRNA sequence and a specific mRNA that leads to the translational repression or degradation of the target mRNA, but also indirectly affect the function of downstream genes through interactions with lncRNAs and circRNAs. The latest studies have highlighted their importance in physiological and pathological processes. MiR‐374 family member are located at the X‐chromosome inactivation center. In recent years, numerous researches have uncovered that miR‐374 family members play an indispensable regulatory role, such as in reproductive disorders, cell growth and differentiation, calcium handling in the kidney, various cancers and epilepsy. In this review, we mainly focus on the role of miR‐374 family members in multiple physiological and pathological processes. More specifically, we also summarize their promising potential as novel prognostic biomarkers and therapeutic targets from bench to bedside.

## 1 INTRODUCTION

ncRNAs represent a newly recognized kind of transcripts that lacks an “open reading frame” (ORF).^1^ Yet, several researches have illustrated possible latent ORFs inside ncRNAs via genome‐wide techniques, which opened arguments regarding the traditionally understood nature of ncRNAs.^2^ In the past decade, ncRNAs have been revealed to be pivotal regulators at different levels by diversified mechanisms, including translation, RNA splicing, DNA replication, gene regulation, genome defence, chromosome structure, bifunctional RNA and as a hormone.^3^

MiRNAs, as a crucial kind of ncRNAs, are a type of small, non‐coding, evolutionarily conserved, single‐stranded, endogenous RNA molecule of approximately 22‐25 nucleotides (nt) in length. They regulate gene expression at the post‐transcriptional level via their “seed sequences,” which hybridize to the 3′‐untranslated region (3′‐UTR), 5′‐UTR and/or CDS (coding sequence) of target mRNAs and lead to degradation or translational inhibition of these target mRNAs.^4^, ^5^ Because miRNAs imperfectly complement their targets, they are able to interact with tens to hundreds of gene products in various signaling pathways to ultimately regulate cell functions. This also indicates the diversity of miRNAs functions.

MiR‐374 family members (including A (a), B (b) and C (c)), as splicing segments of the lncRNA FTX, are located at the X‐inactivation center (Xic) of chromosomes Xq13.2 in humans.^8^ An increasing number of studies have shown that these members participate in a great diversity of physiological and pathological processes. In this review, we mainly summarize the role of miR‐374 family members, a highly conserved miRNA cluster in evolution, in different physiological and pathological processes. More importantly, we also discuss their potential function in the diagnosis and treatment of miR‐374 related diseases, especially cancer.

## 2 THE DELIVERY OF miR‐374 AND ITS ROLE IN DEVELOPMENT

**Figure 1:**
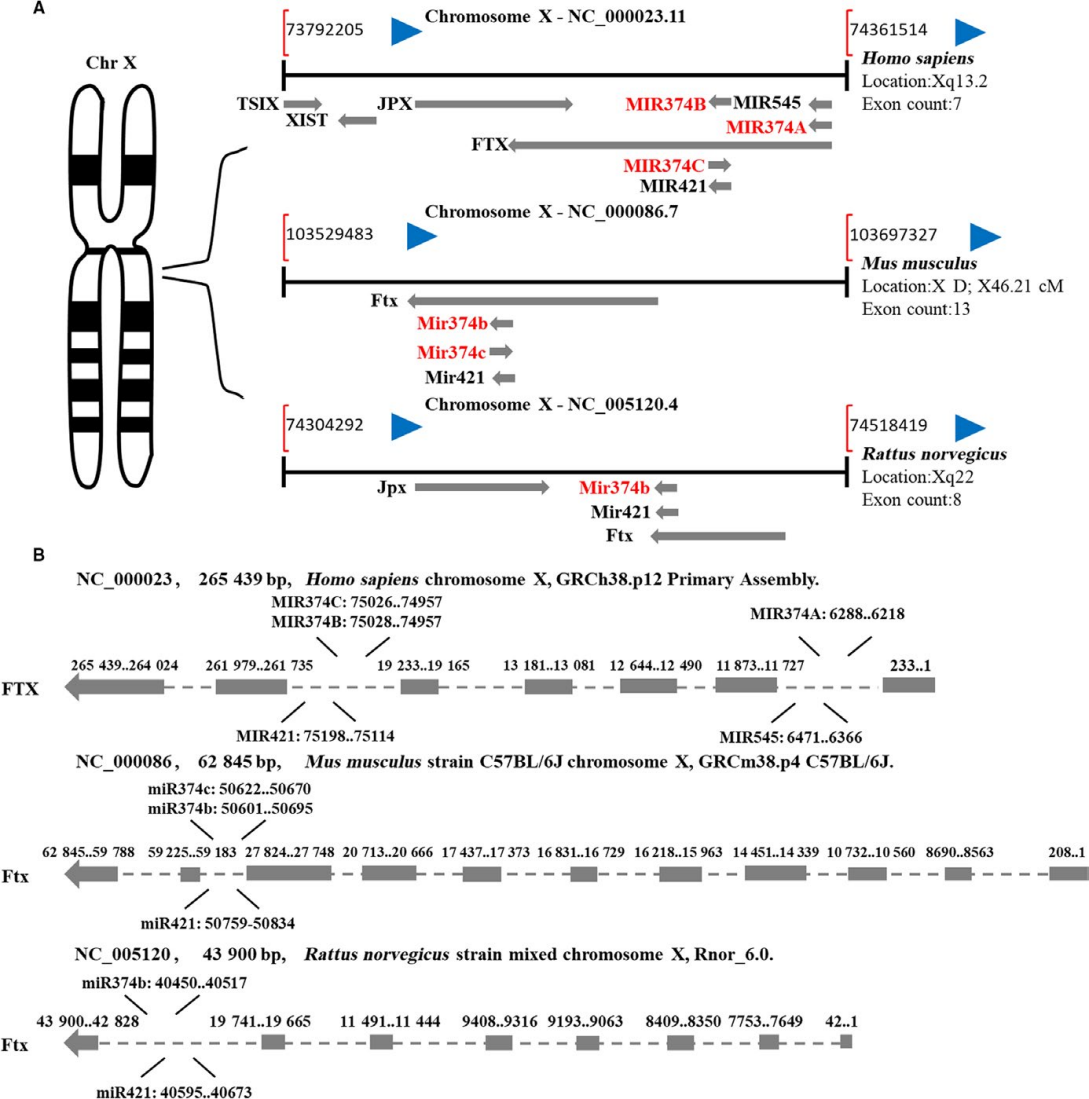
Locus of miR‐374 family members in human, mice and rats. A, Xic of chromosomes X in different species. Red font represents several transcripts of miR‐374 family members. B, Locus of miR‐374 family members in the introns of Ftx in different species (from top to bottom: human, mouse and rat)

In mammalian development, X‐chromosome inactivation (XCI) is well‐known for epigenetic regulation.^9^, ^10^ Mounting studies have shown that female embryos will die without XCI, which corroborates the significance of this epigenetic regulatory mechanism in the development of female embryos.^10^, ^11^ The transcriptional silencing of an X chromosome often corrects an imbalance of X‐linked gene dosage. One cis‐acting element, named Xic, controls and regulates XCI. There are several transcripts that are located in Xic and can escape XCI, including X‐inactive‐specific transcript (XIST), TSIX (TSIX transcript, XIST antisense RNA), JPX (JPX transcript, XIST activator), FTX (FTX transcript, XIST regulator) and miRNAs generated from the FTX sequence by cleavage,^12^, ^13^ which may lead to gender disparity (Figure 1). Overwhelming data have implicated that XIST, the master regulator of X‐inactivation initiation, is a single and central cis‐acting regulator that coordinates imprinted XCI.^11^, ^14^

**Figure 2:**
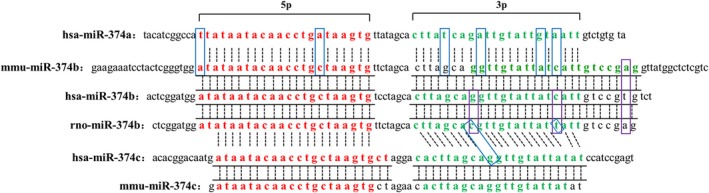
Matching of sequence. Comparison of different species and different fragments of miR‐374 family members. Red font represents miR‐374‐5p. Green font represents miR‐374‐3p. The purple box represents the difference in miR‐374b between different species. The blue box represents the difference between miR‐374 family members. Dotted lines represent the base matches in miR‐374 family members of different species

In addition to XIST, FTX is also one of the most abundant transcripts at the stage of preimplantation embryo and is thought to be a positive regulator of XIST.^13^, ^15^ It is a well‐conserved lncRNA in evolution and includes several conserved miRNAs, such as miR‐374, ‐545 and ‐421, in its introns.^16^ In humans, intron 1 encodes a cluster of 2 miRNAs (MIR‐374A and MIR‐545), which is absent in rats and mice; intron 5 encodes a cluster of 3 miRNAs (MIR‐374B, C and MIR‐421) (Figure 1B). In addition, the sequences of miR‐374 family members are highly conserved in different mammalian species, especially in miR‐374‐5p (Figure 2).

In recent years, studies have shown that these miRNAs, which are located at Xic, are also imprinted and involved in mammalian development and the generation of paternal sperm. For example, Kobayashi et al^10^ reported that miR‐374‐5p and miR‐421‐3p mapped adjacent to Xist, were both predominantly expressed in female blastocysts from the Xp chromosome in F1 blastocysts and were indeed imprinted, as determined by allelic expression analysis. In addition, there is another report^17^ that miR‐374b is markedly decreased in the seminal plasma of azoospermia but is increased in the seminal plasma of asthenozoospermia, as shown by Solexa sequencing and RT‐qPCR analysis. The area under the receiver operating characteristic (ROC) curve of miR‐374b is 0.839 in the 73 azoospermia cases and 0.813 in the 79 asthenozoospermia cases. The authors point out this is markedly higher than for routine biochemical parameters (0.510‐0.622). Their results revealed that miR‐374b level in seminal plasma is a novel, non‐invasive approach for diagnosing male infertility.

## 3 THE ROLE OF miR‐374 IN TUMORIGENESIS

Most likely because of the advancement of preventive examinations and therapeutic interventions, epidemiological studies have provided strong support for the decrease of the mortality rate due to cancer. Nevertheless, the heterogeneous complex of cancers still remains one of the leading causes of premature death worldwide.^18^ Recent reports have verified the presence of ncRNAs, including lncRNA, miRNAs, circular RNAs and so on, in the human cancer.^19^, ^20^ We will summarize the role of miR‐374 family members in the development and progression of different systematic cancers in this section.

### 3.1 miR‐374 and digestive system carcinoma

The digestive system, as one of the eight systems of the human body, is composed of two parts: the digestive tract and digestive glands. The liver, as one of the important digestive glands, plays an indispensable role in body metabolism, including the storage of glycogen, the synthesis of plasma protein, the decomposition of red blood cells, and the detoxification of toxic substances. Meanwhile, because the liver is a fragile organ, poor protection will lead to the decline of normal function and the development of disease (such as hepatitis and hepatocellular carcinoma). In 2017, Bao et al^21^ reported that miR‐374 levels were significantly decreased as HBV‐related liver fibrosis progressed from S0‐S2 to S3‐S4. In addition, they identified that miR‐374 had a highly diagnostic accuracy in discriminating S0‐S2 from S3‐S4 using multivariate logistic regression analysis. In recent years, our research group has been unanimously committed to the study of liver disease. In terms of miRNAs, we have previously revealed that the miR‐545/374a cluster is up‐regulated in HBV‐HCC tissue and is significantly correlated with prognosis‐related clinical features, including histological grade, metastasis and tumor capsule via the observation of 66 pairs of HBV‐related HCC tissue and matched non‐cancerous liver tissue specimens.^8^

Besides observing the role of miR‐374 in liver, some have also investigated its effect on the digestive tract. For example, Wu et al^22^ evaluated the potential value of miR‐374b as a biomarker in colorectal cancer and found that miR‐374b expression was significantly decreased for CRC patients with stage II and stage III disease and may be a novel biomarker for CRC. In addition, Qu et al^23^ more comprehensively analysed the function of miR‐374b from colon cancer tissues to cell lines. First, they showed that the expression of miR‐374b was significantly reduced in colon cancer tissues and cell lines. Second, they investigated the effect and mechanism of miR‐374b and found that its overexpression inhibited cell proliferation and invasion, while the role of its knockdown was exactly opposite. Ultimately, they verified that LRH‐1 is a direct target of miR‐374b via a dual‐luciferase reporter assay, and then they showed that miR‐374b can suppresses the Wnt signaling pathway through LRH‐1 in colon cancer cells.

In recent years, there have also been some reports about the relationship between miR‐374 and gastric cancer. Xie J showed that the expression of miR‐374b‐5p was up‐regulated and conducive to gastric cancer cell invasion and metastasis via inhibiting the expression of RECK.^24^ Ji et al^25^ presented that the up‐regulation of miR‐374 mediated the malignant transformation of gastric cancer‐associated mesenchymal stem cells and represented a novel avenue for gastric cancer therapy with an experimental rat model. In the same year, Sierzega et al found that miR‐374 was abnormally expressed in the primary tissue of gastric cancer, but its expression was not changed in the serum of GC patients.^26^

Taken together, miR‐374 expression was increased in HCC and GC. Additionally, its up‐regulation promoted the malignant transformation of tumor cells. On the contrary, its expression was decreased in CRC. Meanwhile, its down‐regulation repressed colon cancer cell proliferation and invasion. So the role of miR‐374 is consistent in HCC, CRC and GC. Therefore, miR‐374 can be as a novel biomaker in light of its function on the malignant features of digestive system carcinoma, including proliferation, apoptosis, invasion and metastasis.

### 3.2 miR‐374 and other system carcinoma

At present, there are quite sporadic reports of the effect of miR‐374 on tumourgenesis in other systems. For example, miR‐374b is diminished in prostate cancer tissue, and it can be identified as an independent predictor of biochemical recurrence‐free survival by analysing the correlations between its expression and clinical‐pathological features in Chinese patients.^27^ In 2015, Merhautova et al^28^ observed the relationship between miR‐374‐5p and metastatic renal cell carcinoma treated with or without sunitinib by TaqMan low‐density arrays. However, they did not find any connection between either of them. Liao YY have reported that chemokine (C‐C motif) ligand 3 (CCL3) activated MAPK (JNK, ERK, and p38) signaling pathways to reduce miR‐374b expression and promoted VEGF‐A expression and angiogenesis in human osteosarcoma cells.^29^ In 2009, Miko et al found that miR‐374 expression was up‐regulated in primary small cell lung cancer, but they didn't analyze the correlations between its expression and clinical parameters.^30^ The latest report^31^ confirmed that miR‐374 may also participate in the pathogenesis of pituitary gonadotroph adenomas by bioinformatics analysis. However, the author did not discuss the specific mechanism of miR‐374 in this disease.

Regarding skin cancer, Ning et al^32^ revealed that miR‐374c expression was down‐regulated by next‐generation sequencing and RT‐qPCR assay in Merkel cell carcinoma and other cutaneous tumors compared with normal skin. In 2017, Li et al^33^ observed that miR‐374a expression was decreased in skin tissues of human squamous cell carcinoma compared to the normal skin tissue. Meanwhile, they verified through transfection of miR‐374 mimics into A431 and SCL‐1 cells, that miR‐374a down‐regulated the P53 signaling pathway to induce cell apoptosis and inhibit proliferation, migration and invasion by targeting its downstream protein, Gadd45a.

In addition to confirming that miR‐374 influence the development of tumors, there were some reports that proto‐oncogene mutations themselves also affect the expression of miRNAs, including miR‐374. In 2015, García‐Cruz et al^34^ described how p19 affected miRNA by miRNA microarray assays and demonstrated that a p19G12S mutant up‐regulated the expression of miR‐374, miR‐126, miR‐342, miR‐330, miR‐335 and let‐7 in Costello syndrome cell model. Their data suggested the oncogene mutant converted itself into activating status and led to the transduction of the downstream signaling pathways and this may have a sufficiently elementary impact on miRNAs expression to promote the development of numerous cancers.

Taken together, studies on miR‐374 function in cancer have been relatively rare in other system than the digestive system. Only in human osteosarcoma and squamous cell carcinoma of skin tissues, there were detailed reports about miR‐374 activation, its role in several signaling pathways and its downstream targets. The rest of the research has mainly focused on analysing the dependency of the expression of miR‐374 and different clinical‐pathological features.

### 3.3 miR‐374 and chemoradiotherapy of tumor

Presently, cancer treatment mainly includes three types: surgical resection, radiotherapy and chemotherapy. In this section, we mainly discussed miR‐374 and chemoradiotherapy of tumors. In 2016, Schreiber et al^35^ evaluated the potential role of miRNAs in a cisplatin‐resistant pancreatic cancer cell line (BxPC3‐R). They found that 34 miRNAs were up‐regulated and 23 miRNAs were down‐regulated, and then they identified that the down‐regulated miR‐374b was possibly and directly involved in the acquisition of drug‐resistant phenotypes in pancreatic cancer cells with a hidden Markov model algorithm. Meanwhile, miR‐374b overexpression in BxPC3‐R cells recovered cisplatin sensitivity almost to the levels displaying in BxPC3 parental cells. In the same year, Baek et al^36^ first screened the change of miRNAs with microarray analysis in mouse squamous cell carcinoma line NR‐S1, X60 cells (established by irradiating NR‐S1 cells with 10 Gy of X‐ray radiation once every 2 weeks) and C30 cells (established by irradiating NR‐S1 cells with 5 Gy of carbon ion beam radiation once every 2 weeks). They also demonstrated that miR‐374c‐5p and miR‐196a‐5p were down‐regulated. When miR‐374c‐5p was selectively ectopically overexpressed in the human pancreatic cancer cell lines PANC1 and MIA‐PaCa‐2, these cells were sensitized to carbon ion beam radiation, with no change to gamma‐ray sensitivity. Later, Gong et al^37^ demonstrated that the p53/miRNA‐374b/AKT1 signaling pathway may regulate BLM‐induced cell apoptosis of colorectal cancer and ultimately facilitate an improvement in the outcome of chemotherapy in colorectal cancer (CRC). In 2018, Sun et al^38^ verified that the expression of miR‐374b‐5p was significantly reduced in pancreatic cancer tissues and the decreased expression was closely associated with poor progression in patients with pancreatic cancer. Meanwhile, they used multiple human pancreatic cancer cell lines and revealed that miR‐374b‐5p up‐regulation relieved the chemoresistance of pancreatic cancer cells to gemcitabine by targeting several antiapoptotic genes, such as BCL‐2, BIRC3 and XIAP.

Therefore, miR‐374 may be a novel chemosensitizer and/or radiosensitizer and will be a new potential biomarker for deciding the optimal treatment for cancer.

## 4 THE ROLE OF miR‐374 IN CELL GROWTH AND DIFFERENTIATION UNDER PHYSIOLOGICAL CONDITIONS

Cell division, growth and differentiation are common growth processes in organisms. Under physiological conditions, cell division, growth and differentiation are strictly and finely regulated. However, the exact mechanisms of this process are not yet clear.

In recent years, several studies have reported that miRNA‐374 contributes to differentiation and proliferation of different cells in multiple organisms. For example, Dmitriev et al^39^ performed the miRNA expression profile with cultures of CD56^+^ primary myoblasts and myotubes isolated from healthy individuals by an affinity purification procedure. They have demonstrated that a total of 60 miRNAs (including miR‐374) were differentially expressed during serum starvation‐induced myogenic differentiation. However, they did not explore the targets of miR‐374. In 2015, Ma et al^40^ further studied the role of miR‐374 and found that miR‐374b specifically bound to the 3′‐UTR of MRF4 to down‐regulate its expression at both the mRNA and protein level, leading to the negatively regulation of the differentiation of C2C12 myoblasts. In addition, Jee et al^41^ found that the expression of miR‐374‐5p was higher in the proliferative zone (PZ) than the hypertrophic zones. They also identified that primary chondrocytes treated with a PTH/PTHrP receptor agonist, PTH1‐34, induced the expression of miR‐374‐5p. The inhibition of miR‐374‐5p expression decreased chondrocyte proliferation and stimulated hypertrophic differentiation. Meanwhile, Rasheed et al^42^ detected the expression level of miR‐374 in the retina and demonstrated that its expression was up‐regulated from the E12 to the PN1 stage and was later down‐regulated. Nevertheless, this expression pattern was not an inverse with Brn3b during retinal ganglion cell (RGC) development. Subsequently, they confirmed that miR‐374 by itself cannot affect Brn3b expression, but it can work with miR‐23a to synergistically regulate the expression of Brn3b, thereby affecting RGC development.

Accumulated data have shown that miR‐374 regulates cell growth and differentiation not only in rodents but also in poultry. In 2013, Pan et al^43^ showed that dexamethasone‐induced miR‐374a and miR‐374b promoted the differentiation of primary porcine adipocytes by targeting 3′‐UTR of C/EBP‐β. In the same year, Su et al^44^ found that miR‐374 contributed to goat hair production both in entering growth and cessation stages by the analysis of comparative genomics combined with an expression profile assay.

## 5 THE ROLE OF miR‐374 IN KIDNEY DISEASE

Under physiological ranges, extracellular Ca^2+^ regulation is principally maintained by the kidney, as well as the skeleton. A series of studies have demonstrated that Ca^2+^ sensing receptor (CaSR) and claudin (CLDN) are pivotal regulators in renal Ca^2+^ balance. Among them, CaSR monitors circulating Ca^2+^ concentrations by adjusting excretion rates in the kidney.^45^ Moreover, CaSR influences Ca^2+^ transport via alterations of the transepithelial potential and paracellular channel permeability.^46^ The family of CLDNs, as four transmembrane proteins, consist of 27 members, which form paracellularly heteromeric or homomeric channels to allow selective permeation of cations (including Ca^2+^ and Mg^2+^) through the epithelial tight junction.^47^, ^48^ Early in 2009, Hou et al^49^ showed that CLDN16 and CLDN19 are specifically expressed in the thick ascending limb (TAL) of nephrons, where a main percentage of filtered divalent cations (including calcium ions and magnesium ions) are extracellularly reabsorbed (30%‐35% Ca^2+^ and 50%‐60% Mg^2+^). Then in 2012, a study^50^ using biochemical analysis and electrophysiological recordings found that CLDN14 and CLDN16 interacted to involve in renal Ca^2+^ reabsorption. CLDN14 overexpression in kidney epithelial cells impaired paracellular positive ions permeability through the CLDN16/19 heteromeric channel. Given the importance of miRNAs, Gong et al^50^, ^51^ demonstrated that CaSR activation by extracellular Ca^2+^ induced the expression of miR‐374, as a novel microRNA, in TAL cells. Then, the up‐regulated miR‐374 dampened the transcript stability and translation of CLDN14 in a synergistic manner.

Polymeric IgA1 deposited in the mesangial of kidney leads to IgA nephropathy (IgAN). This is one of the most common cause of glomerulonephritis all around the world.^52^, ^53^ Studies showed that IgAN was associated with an increase in B cells number and the incompletely galactosylation of O‐glycans in IgA1. In 2015, Hu et al^55^ demonstrated that miR‐374b expression was higher in B cells compared with controls in IgAN patients. And miR‐374 can target PTEN and Cosmc to increase cell proliferation and the abnormal glycosylation of IgA1.

## 6 THE ROLE OF miR‐374 IN NERVOUS SYSTEM DISEASE

### 6.1 miR‐374 and epilepsy

Epilepsy is one of the common diseases of the nervous system, and its prevalence is second only to stroke. Accumulated data have shown that miRNAs are involved in various neurological diseases, such as Alzheimer's disease (AD),^56^ ischemic tolerance^57^ and Parkinson's disease^58^. But, less is known about miRNAs on epilepsy. In 2014, Moon et al^59^ found that the expression of miR‐374 was significantly decreased in the MDR group versus the control group in a model of mouse pilocarpine‐induced epilepsy. In 2015, Liu et al^60^ induced a rat TLE model with pentylenetetrazol. They also analysed the dysregulated miRNAs in the hippocampus by microRNA expression profiles and found that there were four up‐regulated miRNAs, including miR‐374.

### 6.2 miR‐374 and neurodegeneration

Degenerative changes (including drug‐induced and physiological) in the nervous system seriously threaten people's health. In recent years, research on the degenerative changes of the nervous system by miRNAs has grown. Wang et al^61^ found that PQ‐ and MPTP‐treatment inhibited the expression of miR‐374‐5p (*P* < 0.01) in Neuro‐2a cells. Yet, miR‐374‐5p was not associated with several biological processes including regulation of DNA dependent transcription and RNA metabolic processes in the pathogenesis of Parkinson's disease. Moreover, Manzine^62^ reported that miR‐374 levels were significantly diminished in AD compared with the control group. Furthermore, miR‐374 may directly target relevant AD genes such as BACE1 to regulate the progress of AD.

Amyotrophic lateral sclerosis (ALS) is one of the most common adult onset neurodegenerative diseases with a prevalence of 6‐8 per 100 000.^63^ It is a complex disease with multiple pathogenic mechanisms (including excitotoxicity, oxidative stress, protein aggregation, mitochondrial dysfunction, dysregulated endosomal trafficking, defective axonal transport, dysregulation of RNA processing, and neuroinflammation).^64^ In 2017, Waller et al^65^ reported that miR‐374b‐5p was significantly decreased in patient serum over time compared with 23 sALS and 22 control subjects. And it may be a compensatory role in the degeneration of muscle in ALS and be an attempt to support muscle regeneration and restore a balance by enhancing myoblast differentiation, and thus it could be used as a biomarker to assess treatment efficacy and potentially disease prognosis.

### 6.3 miR‐374 and other diseases in the nervous system

Hypoxic–ischemic encephalopathy (HIE) is a disease defined as ischemic injury caused by hypoxic asphyxia during the perinatal period. Late diagnosis partially leads to high mortality (approximately 15%‐20%) of this diease. Therefore, finding new biomarkers to improve the diagnostic value of neuron‐specific enolase (NSE) and S100B protein is especially important. In 2017, Wang et al^66^ reported that the expressions of miR‐210 and miR‐374a, considered to be two important hypoxia‐associated miRNAs, were down‐regulated in blood samples of HIE newborns compared with those of healthy newborns. Joint analysis of miR‐210, miR‐374a, S100B protein and NSE help to elevate the diagnostic value and prognostic prediction for HIE by the ROC curves assay.

## 7 THE ROLE OF miR‐374 IN CARDIOVASCULAR DISEASE

Studies showed that miR‐374 family members can also regulate the pathophysiological process of cardiovascular disease. Early in 2013, Ward et al^67^ analysed miRNA profiles in different blood subcomponents, such as platelets, PBMCs and plasma, via a high throughput RT‐qPCR system in patients with STEMI or NSTEMI. And they found that miR‐374b‐5p in PBMCs was obviously lower in patients with STEMI than in patients with NSTEMI. Reversely, miR‐374b‐5p in plasma was markedly higher in patients with STEMI compared with NSTEMI. These results suggest the possible involvement of miR‐374b‐5p in ACS subtypes. Under normal circumstances, after patients with ACS are promptly treated, the myocardial cells undergo an ischemia and reperfusion process. Studies of myocardial ischemia‐reperfusion have shown that it does not only brings benefits, but also causes myocardial injury. In 2018, Zhang et al^68^ investigated the effects of miR‐374 on myocardial I/R injury in rat models. Their results demonstrated that miR‐374 relative expression was evidently lowered after reperfusion in the I/R and sevoflurane plus I/R groups compared with the sham group in the myocardium of rats. Compared with the I/R group, miR‐374 relative expression was significantly increased in the sevoflurane plus I/R group in the myocardium of rats. Finally, they found that miR‐374 could alleviate rat myocardial I/R injury by targeting SP1 through activating the PI3K/Akt signal pathway after pretreatment with sevoflurane.

Selenium deficiency has been identified as a causative factor in different kinds of heart failure. Researchers^69^ used microarray hybridization to show that there are five up‐regulated (>5‐fold) miRNAs, which were miR‐374, miR‐16, miR‐199a‐5p, miR‐195 and miR‐30e*, and three down‐regulated miRNAs, which were miR‐3571, miR‐675 and miR‐450a*, in rats with selenium deficiency by. And they verified that miR‐374 expression was the highest among these up‐regulated miRNAs. In the end, they explored that the Wnt/β‐catenin signalling pathway was possibly associated with cardiac dysfunction caused by selenium deficiency. However, they did not confirm the targeting relationship between miR‐374 and Wnt/β‐catenin.

VEGF is a pivotal cytokine that promotes the formation of new blood vessels. However, the VEGF/VEGFR1 signalling pathway regresses cardiac hypertrophy. In contrast, the VEGF/VEGFR2 signaling pathway accelerates cardiac hypertrophy. Lee et al^70^ found that miR‐374 inhibited the VEGFR1 signalling pathway and activated GPCR signalling pathway by targeting the 3′‐UTR of VEGFR1 and cGMP‐dependent protein kinase‐1 to mediated pro‐hypertrophic processes.

In 2016, Licholai et al^71^ found that miR‐374‐5p can maintain vascular integrity by contrasting the different profile of microRNA expression in aneurysmal and unaffected ascending aortic tissue acquired from the same patient. In 2012, Milenkovic^72^ observed that miR‐374* expression increased after mutagenesis in apoE mice compared to wild‐type mice; however, when supplemented with polyphenols in these mice, its expression decreased in apoE. And then they analysed that miR‐374* expression presented negative correlations with AKT1.

## 8 THE ROLE OF miR‐374 IN IMMUNE‐RELATED DISEASE

CD56 is invariably expressed in normal natural killer cell,^73^ a subset of normal T cells and occasionally in T cell acute lymphoid leukemia (T‐ALL).^74^ Studies have implied that CD56 is associated with a poor prognosis in lymphoid tumors, including T‐ALL.^74^ Therefore, in 2013, Gimenes‐Teixeira et al^75^ showed that miR‐374 and miR‐221 were higher in T‐ALL/CD56^+^ than in T‐ALL/CD56^‐^ cells, with 181‐ and 271‐fold relative expression, respectively. Without regard to the expression of CD56, the expression of miR‐374 was at obviously higher levels in leukemic blasts compared with normal peripheral blood thymocytes and T cells.

However, in 2015, Qian et al^76^ further investigated the role and mechanism of how miR‐374 affects T‐cell lymphoblastic lymphoma. They showed that miR‐374b was markedly down‐regulated in the T‐ALL tissues by microRNA microarray analysis, and the down‐regulated miR‐374b was greatly associated with worse survival and higher relapse rates in patients with T‐ALL. miR‐374b overexpression restrained tumorigenicity and cell proliferation, and it accelerated cell apoptosis though targeting Wnt‐16 and AKT1, which led to inhibition of AKT signal pathway.

Acute graft‐versus‐host disease (aGvHD) has a higher mortality rate, the most frequent and serious complication. Expression analysis^77^ identified miR‐374‐5p as significantly down‐regulated and with diagnostic value by ROC analysis in aGvHD.

Moreover, Delić et al^78^ verified that, after female C57BL/6 mice were infected with self‐healing Plasmodium chabaudi malaria, hepatic miR‐374* expression was down‐regulated. In addition, Uribe et al^79^ found that miR‐374a‐5p expression was up‐regulated in porcine intestinal mucosa infected with Salmonella Typhimurium by Microarray hybridization and analysis and RT‐qPCR assay.

## 9 THE ROLE OF miR‐374 IN OTHER DISEASES

Bhargava et al^80^ found that miR‐374 affected the function of rat AT2 epithelial cells during hyperoxic stress and recovery through three possible targets, including actinin alpha 4, actinin alpha 1, and Na, K‐ATPase.

In addition, some have directly observed the relationship between miR‐374 and other critical molecules in different cell lines. For example, Tasharrofi et al^81^ wanted to investigate whether miR‐374a can inhibit Fas‐induced apoptosis in human primary retinal pigment epithelial (RPE) cells by targeting Fas during oxidative conditions. Their results confirmed that miR‐374a indeed prevented Fas up‐regulation by binding with its 3′‐UTR to enhance RPE cells survival and protect the cells against oxidative stress. Unterbruner et al^82^ found that miR‐374a‐5p regulate not only the expression of ubiquitin ligase MID1 by binding to the 3′‐UTR of the MID1 mRNA but also the mTOR signaling pathway. Therefore, given that dysregulation of MID1 expression is closely associated with multiple diseases including cancer, midline malformation syndromes and neurodegenerative diseases, miR‐374a‐5p could serve as a potential drug target for future therapy development.

## 10 CONCLUSIONS AND PERSPECTIVES

In conclusion, as shown in Table 1, although there are some studies on the impact of miR‐374 family members on various diseases, especially cancer, their expression in different pathophysiologies are not the same. In addition, investigations on miR‐374 family members are relatively superficial, both physiologically and pathologically, and mainly include the detection of miR‐374 expression performed by microarrays or RT‐qPCR assays and the analysis of correlation between miR‐374 expression and cell apoptosis, invasion, metastasis and relapse, etc. There are relatively few studies on their targets. To date, the targets of miR‐374 family members chiefly include: AKT, VEGF, PTEN, Wnt and Fas signalling pathways. Therefore, the mechanism of miR‐374 in different cells or disease models needs further exploration and verification.

**Table 1:** Comparison of miR‐374 family members under different pathophysiological conditions

Disease	Family members	Species	Tissue and/or cell	Expression	Targets or pathway	Relationship with disease or clinical significance	Time	Ref.
Imprinted gene cluster	miR‐374‐5p	Mouse	Blastocysts	Up	/	miR‐374‐5p were imprinted	2013	10
Male infertility	miR‐374b	Human	Seminal plasma	Down	/	Azoospermia	2011	16
				Up		Asthenozoospermia		
HBV‐related liver fibrosis	miR‐374	Human	Serum	Down	/	As a noninvasive diagnostic biomarker	2017	21
HBV‐related hepatocellular carcinoma	miR‐374a	Human	HBV‐HCC tissue and HCC cell lines Bel‐7402, HepG2, HepG2215	Up	/	Correlated with histological grade, metastasis and capsule of HCC	2015	8
Colorectal cancer	miR‐374b	Human	Colon cancer tissues	Down	/	As a biomarker of CRC	2015	22
	miR‐374b	Human	Colon cancer tissues and cell lines HT29, HCT116, SW480 and SW620	Down	LRH‐1	Inhibited colon cancer cell proliferation and invasion	2018	23
Gastric cancer	miR‐374b‐5p	Human	Gastric carcinoma cell line MGC‐803, SGC‐7901 and the normal human gastric epithelial cell line GES‐1	Up	RECK	Promoted gastric cancer cell invasion and metastasis	2014	24
	miR‐374	Rat	Wistar rats and primary MSCs	Up	/	Malignant transformation of gastric cancer associated mesenchymal stem cells (MSC)	2017	25
	miR‐374a‐5p	Human	Blood and tissue samples	Up	/	Evaluation of serum microRNA biomarkers for gastric cancer	2017	26
Prostate cancer	miR‐374b	Human	Prostate cancer tissue	Down	/	Correlation with clinical features of prostate patients	2013	27
Renal cell carcinoma	miR‐374‐5p	Human	Tissue samples with or without sunitinib	/	/	Didn't found any connection	2015	28
Osteosarcoma	miR‐374b	Human, mouse	Tumor tissue and osteosarcoma cell lines MG‐63, U‐2 OS and endothelial progenitor cell (EPC)	Down	CCL3/MAPK/miR‐374b/VEGF‐A	CCL3 promoted angiogenesis by regulating miR‐374b/VEGF‐A axis	2016	29
Small cell lung cancer	miR‐374	Human	Tissue samples and cell lines HTB‐172, HTB‐184, HTB‐119	Up	/	/	2009	30
Pituitary gonadotroph adenomas	miR‐374	Rat	Pituitary tissue	Up	/	MiR‐374, ‐153, ‐145 and ‐33 may have regulated the DEGs.	2018	31
Skin cancer	miR‐374c	Human	Tissue samples and MCC cell line MS‐1	Down	/	/	2014	32
	miR‐374a	Human	Skin SCC samples and normal skin cells and SCC skin cell line A431 and SCL‐1	Down	Gadd45a (downstream protein of P53 signaling pathway)	Induced cell apoptosis and inhibitd proliferation, migration and invasion	2017	33
Mutant of p19 and p21 H‐Ras proteins	miR‐374	Human, mouse	HeLa cells and murine embryonic fibroblasts (MEFs)	Up	/	/	2015	34
Cisplatin resistant	miR‐374b	Human	Cisplatin‐resistant pancreatic cancer cell line BxPC3‐R	Down	/	Acquisition of drug‐resistant phenotype of pancreatic cancer cell	2016	35
Carbon ion beam radiotherapy	miR‐374c‐5p	Mouse, Human	Mouse squamous cell carcinoma line NR‐S1, human pancreatic cancer cell lines PANC1 and MIA‐PaCa‐2	Down	/	Increased the sensitivity of both PANC‐1 and MIA‐PaCa‐2 cells to carbon ion beam irradiation	2016	36
Colorectal cancer	miR‐374b	Human	Colorectal cancer cell lines HCT116 and HT29	Up	p53/miRNA‐374b/AKT1	Regulate BLM‐induced cell apoptosis, and improved the outcome of chemotherapy in CRC	2017	37
Chemotherapeutic resistance of pancreatic cancer	miR‐374b‐5p	Human	Pancreatic cancer cell lines BxPC‐3, PANC‐1, AsPC‐1, SW1990, Capan‐1, Capan‐2, CFPAC‐1 and MIA PaCa‐2; pancreatic cancer tissues	Down	Antiapoptotic proteins: BCL‐2, BIRC3 and XIAP	The decreased expression of miR‐374‐5p was associated with poor overall and progression free survival. The up‐regulation of miR‐374b‐5p ameliorated the chemoresistance of pancreatic cancer cells to gemcitabine.	2018	38
Myogenic differentiation	miR‐374	Human	Primary myoblasts and immortalized myoblasts (iMyo)	Up	/	/	2013	39
C2C12 myoblasts differentiation	miR‐374b	Human	C2C12 cells	Down	MRF4	Suppressed myoblast differentiation	2015	40
Growth plate of cartilage	Mir‐374‐5p	Rat	Primary chondrocytes (PZ)	Up	/	Promoted proliferation and inhibited hypertrophic differentiation	2018	41
			Hypertrophic chondrocytes (HZ)	Down		Inhibited proliferation and promoted hypertrophic differentiation		
Retinal ganglion cell development	miR‐374b	Mouse	E14 embryos, RGC‐5 cells	Up (E12 ‐PN1 stage)	Brn3b	miR‐23a alone or in combination with miR‐374 could attribute to the biphasic expression pattern of Brn3b, thereby affecting the RGC development, but miR‐374 by itself cannot regulate the expression of Brn3b	2014	42
				Down (later on)				
Adipocytes differentiation	miR‐374a and ‐374b‐5p	Porcine	Primary porcine preadipocyte	Up	C/EBP‐β	Promoted differentiation of primary porcine adipocytes	2013	43
Hair production	miR‐374b	Goat	Longissimus dorsi, leg and skin tissue	Up	/	Pushing secondary hair follicle activity changes from catagen to telogen	2015	44
Ca^2+ ^homeostasis	miR‐374b	Human, mouse	Wild‐type and CLDN14 KO mice, primary cultures of mouse TAL cells, mouse MKTAL cells, human HEK293 cells	Up	Ca^2+^/CaSR/miR‐374/CLDN14	Renal Ca^2+^ reabsorption	2012	50
							2014	51
IgA nephropathy	miR‐374b	Human	Renal tissue and CD19^+^ B cells or DAKIKI cells	Up	PTEN and Cosmc	Increase cell proliferation and abnormal glycosylation of IgA1	2015	55
Drug resistant epilepsy	miR‐374	Mouse	Brain tissue	Down	/	/	2014	59
Pilocarpine induced epilepsy	miR‐374‐3p	Rat	Hippocampus tissue	Up	/	/	2015	60
PQ or MPTP treatment induced dopaminergic neurodegeneration	miR‐374‐5p	Mouse	Neuro‐2a cells	Down	/	/	2018	61
Alzheimer's disease	miR‐374	Human	Tissues and cell lines (neuroblastoma SH‐SY5Y cells)	Down	BACE1	As potential biomarker to improve AD diagnosis	2018	62
Amyotrophic lateral sclerosis	miR‐374b‐5p	Human	Serum	Down	/	Promote myoblast differentiation to compensate for the muscle degeneration associated with ALS	2017	65
Hypoxic–ischemic encephalopathy	miR‐374a	Human	Serum of umbilical cord blood	Down	/	MiR‐374a could help to elevate the diagnostic value and prognostic prediction of S100B and NSE for HIE	2017	66
Acute Coronary Syndrome	miR‐374b‐5p	Human	PBMCs	Down	/	STEMI as compared with NSTEMI	2013	67
			Plasma	Up				
Myocardial I/R	miR‐374	Rat	Left ventricular tissue, HEK‐293T cells, cardiomyocytes	Down (I/R)	PI3K/Akt/miR‐374/SP1	Exerted protective effects by inhibiting SP1 through activating the PI3K/Akt pathway in rat models pretreated with sevoflurane	2018	68
				Up (sevoflurane + I/R)				
Cardiac dysfunction of selenium deficiency	miR‐374	Rat	Heart tissue	Up	Wnt/β‐catenin signaling pathway	Mainly associated with miR‐374	2015	69
Cardiac Hypertrophy	miR‐374‐3p	Rat	Neonatal rat ventricular myocytes, Isolated cardiomyocytes	Down	VEGFR1 and PKG‐1	Activated cardiac hypertrophy via activation of the Ca^2+^ signaling pathway	2017	70
Aneurysmal	miR‐374a‐5p	Human	Tissue samples of ascending aorta	Up	/	Maintained vascular integrity	2016	71
Polyphenols feeding	miR‐374*	Mouse	Livers tissue of wild‐type or apoE‐deficient mice	Down	AKT1	Identified as being commonly modulated by these polyphenols	2012	72
T cell acute lymphoid leukemia	miR‐374	Human	Bone marrow samples, thymocytes and peripheral blood T‐cells	Up	/	/	2013	75
T‐cell lymphoblastic lymphoma	miR‐374b	Human	T‐LBL tissue samples, T‐cell lines (Jurkat and SUP‐T1)	Down	Wnt‐16 and AKT1	Associated with worse survival and higher relapse rate in patients with T‐ALL	2015	76
Acute graft‐versus‐host disease	miR‐374b‐5p	Human	Serum	Down	/	Had diagnostic value by ROC analysis	2017	77
Mice infected with self‐healing P. chabaudi malaria	miR‐374*	Mouse	Sera and livers	Down	/	/	2011	78
Porcine infected with Salmonella Typhimurium	miR‐374a‐5p	Porcine	Intestinal mucosa tissue	Up	/	/	2016	79
Hyperoxic stress and recovery induced lung injury	miR‐374	Rat	AT2 epithelial cells	/	Actinin alpha 4, actinin alpha 1, and Na,K‐ATPase	/	2013	80
Age‐related macular degeneration	miR‐374a	Human	Primary human RPE cells	/	Fas	miR‐374a could prevent Fas up‐regulation under oxidative conditions to improve survival of human RPE cells	2017	81
/	miR‐374a‐5p	Human	HEK293T, derivative of HEK293T stably expressing HTT‐exon 1 with 51 CAG‐repeats	/	E3 ubiquitin ligase MID1	/	2018	82

HCC, Hepatocellular Carcinoma; CRC, colorectal cancer; LRH‐1, Liver receptor homolog‐1; GC, gastric cancer; RECK, reversion‐inducing cysteine‐rich protein with Kazal motif; MSC, mesenchymal stem cells; PC, prostate cancer; EPC, Endothelial progenitor cell; SCLC, small cell lung cancer; MCC, Merkel cell carcinoma; SCC, squamous cell carcinoma; DEGs, Differentially expressed genes; BCL2, B‐cell lymphoma 2; BIRC3, Baculoviral IAP Repeat Containing 3; XIAP, X‐linked inhibitor of apoptosis.; TAL, thick ascending limb; RGC, Retinal Ganglion Cell; IgAN, IgA nephropathy; ALS, amyotrophic lateral sclerosis; ACS, acute coronary syndrome; I/R, Ischemia‐Reperfusion T‐ALL, T cell acute lymphoid leukemia; T‐LBL, Lymphoblastic lymphoma of T‐cell lineage; IgAN, IgA nephropathy; HIE, Hypoxic–ischemic encephalopathy; aGvHD, Acute graft‐versus‐host disease; AMD, Age‐related macular degeneration; PZ, proliferative zone; HZ, hypertrophic zones; NRVMs, Neonatal rat ventricular myocytes; RPE, retinal pigment epithelial.

With regard to the sundry possibilities for the diagnosis and treatment of the miR‐374 family members in diseases, we summarize as follows: (a) the diagnostic role of miR‐374 family members. In view of the relationship between the expression of these members and multiple diseases, we can measure their expressions, as a novel biomarker, in serum and/or tissue from patients to assess the likelihood of illness and the prognosis of disease, especially in cancer. (b) The therapeutic role of miR‐374 family members. In this aspect, we can overexpress or knockdown these members themselves by a variety of methods, such as mimics or inhibitor, ago‐ or antago‐miRNAs, over‐expressed or interfering vectors, transgenic or knocking gene and so on. In addition, we can also affect their roles by regulating their targets. However, we found that there were only a small number of papers revealed the targets of miR‐374 family members under different pathological and physiological conditions. This will also be a deficiency in this area.

Therefore, there is still a long way to go until they are used in disease prediction and targeted therapy. So we should accelerate the process of translation of preclinical results into clinic and make them into phase I and II trials to guide clinical diagnosis and treatment. But, this will be a large challenge and hard to pursue in the future. Indeed, the safety (activation of viral delivery systems to immune response, off‐target effect of ncRNAs, competition with endogenous miRNAs and multi‐targeting of miRNAs) of ncRNAs, as clinical therapeutic targets, needs to be established with certainty.

## CONFLICTS OF INTEREST

None.
